# Mothers’ nonstandard work schedules and adolescent obesity: a population-based cross-sectional study in the Tokyo metropolitan area

**DOI:** 10.1186/s12889-021-10279-w

**Published:** 2021-01-28

**Authors:** Yuko Kachi, Aya Abe, Hisashi Eguchi, Akiomi Inoue, Akizumi Tsutsumi

**Affiliations:** 1 Department of Public Health, Kitasato University School of Medicine, Kanagawa, Japan; 2 School of Humanities and Social Sciences, Tokyo Metropolitan University, Tokyo, Japan; 3 Department of Mental Health, Institute of Industrial Ecological Sciences, University of Occupational and Environmental Health, Fukuoka, Japan; 4 Institutional Research Center, University of Occupational and Environmental Health, Fukuoka, Japan

**Keywords:** Adolescent, Obesity, Nonstandard work schedule, Mother, Tokyo

## Abstract

**Background:**

Many wage earners in developed countries have irregular shift patterns and work evenings, nights, and weekends. Some studies have demonstrated that the nonstandard work schedules of parents have adverse effects on their children’s weight, specifically leading to or worsening obesity. However, no such study has been conducted in adolescents of high school age. This study examined the association between mothers’ nonstandard work schedules and adolescent obesity.

**Methods:**

A cross-sectional study of adolescents aged 16–17 years (*n* = 1743) used paired matches of self-administered questionnaires for adolescents and their mothers from Tokyo, Japan. Obesity was defined by International Obesity Task Force cut-offs. Nonstandard work schedules were defined as working early mornings, nights, overnights, or weekends. Chi-square tests were conducted to examine the association between the mothers’ work schedules and their adolescent children’s poor lifestyles, overall and stratified by income levels. Then, logistic regression analyses were conducted to examine the association between mothers’ work schedules and obesity of their adolescent children, overall and stratified by income levels.

**Results:**

Overall, 6.5% of adolescents had obesity. The prevalence of obesity was higher among adolescents from low-middle income groups (8.2%) than that among those from high-income groups (5.9%). No clear patterns were found between mothers’ work schedules and adolescents’ poor lifestyles when stratified by income levels. Mothers’ nonstandard work schedules were significantly associated with adolescent obesity (odds ratio [OR]: 1.56, 95% confidence interval [CI]: 1.02–2.40). However, this association was not significant after adjustment for confounders. After stratification by income levels, mothers’ nonstandard work schedules significantly associated with their children’s obesity (OR: 2.93, 95% CI: 1.45–5.92) only in high-income groups. This association remained after adjustment of the adolescents’ lifestyles and their mothers’ working hours.

**Conclusions:**

Our results suggest that mothers’ nonstandard work schedules have adverse effects on adolescent obesity only in high-income families. Low-middle income families experienced higher levels of adolescent obesity, regardless of the mothers’ work schedules. Policy makers should consider mothers’ work schedules as a factor in preventing adolescent obesity.

**Supplementary Information:**

The online version contains supplementary material available at 10.1186/s12889-021-10279-w.

## Background

Adolescent obesity is a major public health concern worldwide [1]. It not only leads to physical and psychological comorbidities [2], but also to adult obesity [3], and obesity-related mortality [4]. The prevalence of adolescent obesity in Japan is lower compared to many other countries; however, it greatly increased between the late 1970s and early 2000s, and has since shown no sign of declining [5]. Notably, this increase corresponded to another significant change that during this time period, that of an increasing number of mothers joining the workforce. Between 1970 and 2016, the workforce participation rate of women aged 15–64 years increased from 53 to 68% [6]. It is hypothesized that mothers’ employment, especially with long working hours and nonstandard work schedules (NSWS), affects children’s obesity, even in higher age brackets.

The etiology of adolescent obesity is multifactorial and influenced by parental factors and adolescent lifestyle habits [7]. Long working hours of parents have consistently been associated with childhood obesity in several developed countries [8, 9]. An emerging body of research further suggests that parental NSWS can negatively affect childhood obesity [10]. NSWS refers to schedules in which the majority of working hours fall outside the standard daytime Monday-to-Friday work week, although this varies across studies [10]. NSWS generally include early mornings, evenings, nights, rotating shifts, and weekends. In a 24-h, 7-day-a-week economy, many adults have NSWS in Japan as well as in other developed countries [11, 12].

To the best of our knowledge, only four studies to date have evaluated the association between parental NSWS and childhood obesity or body mass index (BMI), and these were conducted in the USA [13, 14], Australia [15], and Germany [16]. Of these, two studies were based on longitudinal data [13, 14]. One study found no significant association between parental NSWS and BMI among American children [14]. These studies included children of upper elementary [14–16] and junior high school ages [13]. In addition, one study suggested that the association between parental NSWS and child BMI was stronger among children living in lower-income households [13]. Current research and evidence on this subject are limited, inconclusive, and absent for high school age adolescents.

Japan is a society where the male breadwinner model has prevailed deeply [17]. The labor force participation rate of Japanese women is below 50%: which means that many women are full-time housewives. However, even married women need to work for a living. Gender role stereotypes have led men to devote themselves entirely to their professions, and household chores and caregiving are considered women’s work. Employed women are forced to undertake household chores in addition to their professional careers. Many women quit formal work temporarily after marriage or childbirth, which results in an M-shaped curve in their work participation rate when stratified according to age group. The living conditions of employed women with children who need care are expected to be challenging unless enough support is provided.

We therefore examined the association between mothers’ work schedules and prevalence of obesity among adolescents aged 16–17 years, hypothesizing that mothers’ NSWS had adverse effects on obesity. We also examined whether the association may differ between household income levels. Since mothers’ employment may affect child obesity [16], and as there are many full-time housewives in Japan, we included unemployed mothers (i.e., full-time housewives) in our analysis. The study of obesity in adolescents of high school age is significant, as a past study reported that obesity at this age is more strongly correlated with adult obesity than obesity at elementary and junior high school ages [3].

## Methods

### Data source and study sample

We used data from the Survey of Child Living Conditions, a cross-sectional study conducted by the Tokyo Metropolitan Government Bureau of Social Welfare and Public Health in Japan [18]. Eligible participants were all adolescents aged 16–17 years and their parents living in four local districts in Tokyo. Adolescents were identified using the resident registers. Two separate self-administered questionnaires for adolescents and their parents were mailed to 6848 households between August 5, 2016 and September 7, 2016. The questionnaires were returned by 2605 adolescents (38.0%) and 2649 parents (38.7%). Parental and adolescent questionnaires were returned in the same envelope, and we selected 2576 pairs for analysis which had been completed by both the adolescents and their parents. We excluded pairs with any of the following criteria: absence of mothers (*n* = 223); respondents not being mothers (*n* = 384); and missing data on variables of interest excluding household income (*n* = 226). A total of 1743 pairs were included in the final analysis. The mothers excluded from the analysis did not differ from the mothers included in the analysis in terms of the prevalence of obesity among their children; however, they had less NSWS and had more missing on income. We obtained permission from the Tokyo Metropolitan Government to use the survey data. Ethics approval for the present study was not required because this was a retrospective analysis of public surveillance data that is free of personally identifiable information.

### Outcome: obesity

Data on height and weight were obtained from the questionnaire which asked adolescents to provide their height (to the nearest cm) and weight (to the nearest kg). Obesity was defined according to the International Obesity Task Force (IOTF) age- and sex-specific BMI cut-offs for overweight [19]. IOTF criteria for overweight are based on identifying the childhood BMI thresholds that correspond to adult BMI thresholds of 25 kg/m^2^. According to the Japanese Society for the Study of Obesity [20], obesity in Japanese adults is defined as a BMI of 25 or more, which corresponds to the WHO standard of overweight plus obese. Thus, we used the cut-off value for overweight as the cut-off value for obesity.

### Predictor: mothers’ work schedules

Mothers were asked if they usually worked the following work shifts: (1) early morning (5–8 am); (2) night (2–10 pm); (3) overnight (10 pm–5 am); (4) Saturday; (5) Sunday or holiday; (6) others; and (7) only standard work hours. For this question, mothers could provide multiple answers. We defined NSWS as schedules other than “only standard work hours.” If mothers were full-time housewives, they were categorized as “not employed.”

### Confounders

Data on potential confounders was obtained from adolescents’ questionnaires and included sex (male, female), type of high school (ordinary, evening or others, not attending), and employment status (employed, not employed). Adolescents’ employment included part-time work, temporary work, and full-time work. Data on maternal confounders was obtained from mothers’ questionnaires and included age (30–39, 40–49, ≥ 50 years), psychological distress (absent, present), household income (low–middle [< 7 million JPY (66 thousand USD)], high [≥ 7 million JPY (66 thousand USD)], missing), living with grandparents of the adolescent (yes, no), living with siblings of the adolescent (yes, no), and father’s work schedules (standard, nonstandard, not employed, no father [single mother household]). Mothers’ psychological distress was measured using the Japanese version of the K6, a self-rated, six-item scale, the reliability and validity of which has been confirmed in the Japanese population [21, 22]. The K6 asks how often respondents have experienced symptoms of nonspecific psychological distress during the past 30 days (e.g., “How often did you feel nervous?”). Each item was scored on a five-point Likert scale (0 = none of the time; 4 = all of the time). The total scores ranged from 0 to 24, with higher scores indicating greater psychological distress. We used a cut-off point of ≥5 to define the presence of mild psychological distress, which was found to be optimal in a validation study conducted in Japan [23]. The annual household income, before taxes, social security premiums, and social transfers, was measured by the 13 response options from zero yen to ≥900 million yen. Questions without a response to household income were categorized as “missing.” The remainder were dichotomized into two groups, “low–middle” and “high,” using the 2016 median income (about 700 million yen) for households with children under 18 years of age [24].

### Adolescents’ lifestyle habits

The following adolescent lifestyle habits were considered as potential mediators: screen time, physical activity, eating-out frequency, and the level of understanding of classes. These were measured by adolescents’ questionnaire responses. Screen time was measured by asking adolescents to report the daily amount of time spent playing games and watching television or using the Internet, and it was dichotomized as ‘recommended’ (< 2 h/d) or ‘excessive’ (≥ 2 h/d), based on the American Academy of Pediatrics’ international guidance on limiting pediatric screen time [25]. Physical activity was assessed by asking adolescents how frequently they participate in sport or physical activity (e.g., cycling [including commuting to and from school], soccer, baseball, karate, kendo, gymnastics, ballet, and swimming) for over 30 min each week. Responses were dichotomized as “physically inactive” (< 1 d/week) versus “active” (≥ 1 d/week). The frequency of eating dinner at restaurants on weekdays was dichotomized as < 1 d/week and ≥ 1 d/week. The level of understanding of classes was measured with five options and dichotomized as “high” (always/usually/sometimes) and “low” (occasionally/rarely).

### Statistical methods

We first described the participants’ characteristics against their mothers’ work schedules. Next, we calculated the percentages of adolescents’ obesity and poor lifestyles against their mothers’ work schedules and household income levels. Logistic regression analysis was then used to compare adolescents’ obesity in the control group (adolescents whose mothers only work standard work schedules: SWS) and the adolescents with obesity whose mothers have NSWS or are not employed, overall and stratified by household income for both groups. We constructed three models: model 1, including confounders except for fathers’ work schedule; model 2, including all confounders; and model 3, including adolescents’ lifestyle habits plus model 2. Finally, two sensitivity analyses were performed. The first analysis presented NSWS as five categories (early morning, night, overnight, Saturday, Sunday or holiday) to examine whether each work shift was associated with adolescents’ obesity, overall and stratified by adolescents’ sex. The second analysis was restricted to employed mothers only and included mothers’ working hours (< 40 h/week, 40–59 h/week, ≥ 60 h/week, missing) in the model to examine whether mothers’ NSWS was associated with adolescents’ obesity independent of work hours. All statistical tests were two-sided, with a 5% significance level, and analyses were conducted using SAS version 9.3 for Windows (SAS Inc., Cary, NC, USA).

## Results

### Characteristics of adolescents

Table 1 demonstrates that 36.6% of mothers had NSWS. Compared to mothers working SWS, mothers with NSWS were more likely to have long working hours, lower household income, no father in the household or father in the household also with NSWS, and adolescents in employment.

**Table 1:** Characteristics of adolescents with their mothers’ work schedule (*N* = 1743)

	Mother’s’ work schedule						
	Standard		Nonstandard		Not employed		
	N	%	N	%	N	%	*p*-value^a^
**All**	739	(42.4)	637	(36.6)	367	(21.1)	
**Child’s characteristics**							
Sex							
Boy	375	(50.7)	316	(49.6)	189	(51.5)	0.832
Girl	364	(49.3)	321	(50.4)	178	(48.5)	
Type of high school							
Ordinary	688	(93.2)	593	(93.4)	338	(92.1)	0.484
Evening or other	45	(6.1)	34	(5.4)	27	(7.4)	
Not attending	5	(0.7)	8	(1.3)	2	(0.5)	
Employment status							
Employed	120	(16.2)	133	(20.9)	39	(10.6)	< 0.001
Not employed	619	(83.8)	504	(79.1)	328	(89.4)	
**Mother’s characteristics**							
Age							
30–39 years	18	(2.4)	16	(2.5)	7	(1.9)	0.474
40–49 years	522	(70.6)	439	(68.9)	242	(65.9)	
≥ 50 years	199	(26.9)	182	(28.6)	118	(32.2)	
Work hours							
< 40 h/week	535	(72.4)	356	(55.9)	–	–	< 0.001
40–59 h/week	181	(24.5)	242	(38.0)	–	–	
≥ 60 h/week	7	(1.0)	31	(4.9)	–	–	
Missing	16	(2.2)	8	(1.3)	–	–	
Psychological distress (K6 ≥ 5)							
Absent	510	(69.0)	406	(63.7)	244	(66.5)	0.118
Present	229	(31.0)	231	(36.3)	123	(33.5)	
**Household characteristics**							
Household income							
Low–middle (< 7 million JPY)	238	(32.2)	281	(44.1)	102	(27.8)	< 0.001
High (≥ 7 million JPY)	386	(52.2)	262	(41.1)	177	(48.2)	
Missing	105	(15.6)	94	(14.8)	88	(24.0)	
Living with grandparents							
Yes	88	(11.9)	89	(14.0)	42	(11.4)	0.395
No	651	(88.1)	548	(86.0)	325	(88.6)	
Living with siblings							
Yes	603	(81.6)	507	(79.6)	282	(76.8)	0.174
No	136	(18.4)	130	(20.4)	85	(23.2)	
Father’s work schedule							
Standard	335	(45.3)	164	(25.8)	185	(50.4)	< 0.001
Nonstandard	303	(41.0)	327	(51.3)	136	(37.1)	
Not employed	9	(1.2)	15	(2.4)	14	(3.8)	
No father (single mother household)	92	(12.5)	131	(20.6)	32	(8.7)	

^a^Chi-square test

### Adolescents’ obesity and lifestyle habits

**Fig. 1:**
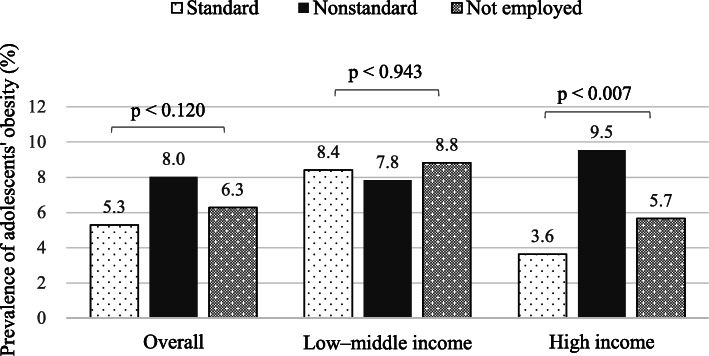
Mothers’ work schedules and adolescents’ obesity: overall and associated with income levels. The chi-square test is used to compare the prevalence of obesity associated with mothers’ work schedules

Overall, 6.5% of adolescents had obesity. The prevalence of obesity did not differ significantly with mothers’ work schedules (Fig. 1). Adolescents from low-middle income families had a higher prevalence of obesity (8.2%) than that in adolescents from high-income families (5.9%). When stratified by income level, a significant difference was observed only in the high-income group, with a higher prevalence of obesity among adolescents whose mothers had NSWS compared to those whose mothers had SWS.

**Fig. 2:**
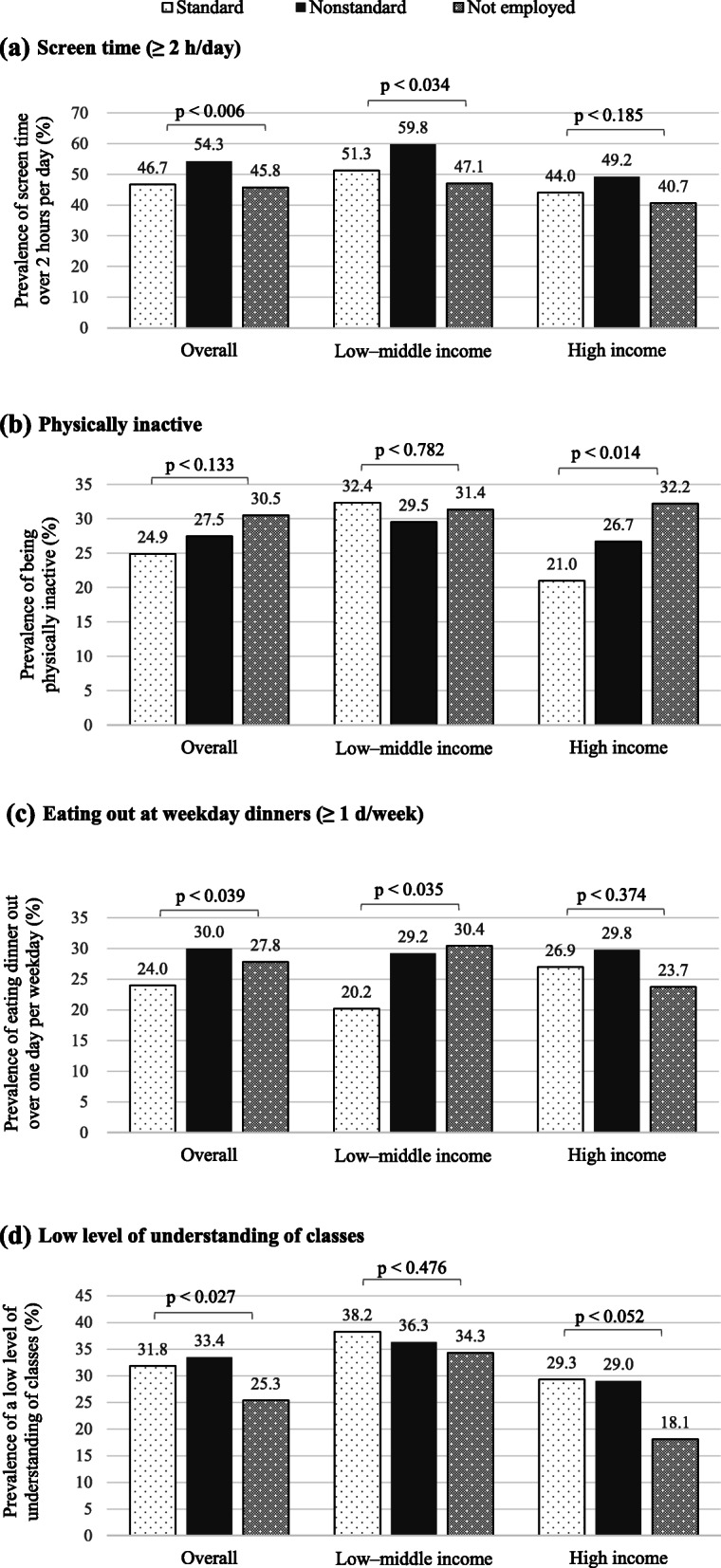
Mothers’ work schedules and adolescents’ lifestyles, overall and associated with income levels. The chi-square test is used to compare the prevalence of obesity associated with mothers’ work schedules. **a** Screen time (≥ 2 h/day). **b** Physically inactive. **c** Eating out at weekday dinners (≥ 1 d/week). **d** Low level of understanding of classes

Regarding adolescents’ lifestyle habits (Fig. 2), adolescents whose mothers had NSWS were more likely to have high screen time, high eating-out frequency at weekday dinners, and low level of understanding of classes than those whose mothers had SWS. However, there was variation when lifestyle habits were stratified by household income. In the low–middle income group, the percentages of high screen time and high eating-out frequency at weekday dinners were higher among adolescents whose mothers had NSWS than those whose mothers had SWS. In the high-income group, the ‘physically inactive’ percentage was higher among adolescents whose mothers had NSWS than those whose mothers had SWS.

### Mothers’ NSWS and adolescents’ obesity

Table 2 shows the odds ratios (OR) and 95% confidence intervals (CI) of adolescents’ obesity. Overall, mothers’ NSWS was significantly associated with adolescents’ obesity compared with mothers who had SWS (OR: 1.56, 95% CI: 1.02–2.40). However, this association was not significant after adjustment of confounders. After stratification by income level, mothers’ NSWS was significantly associated with adolescents’ obesity only in high-income groups (OR: 2.80, 95% CI: 1.43–5.50). The OR remained significant after adjustment for confounders except for fathers’ work schedule (OR: 2.83, 95% CI: 1.41–5.69), and after additional adjustments for fathers’ work schedule (OR: 2.93, 95% CI: 1.45–5.92). Further adjustment of adolescents’ lifestyle habits did not substantially change the OR. However, for the low–middle income group, no significant associations were observed between mothers’ work schedule and adolescents’ obesity.

**Table 2:** Association between mothers’ work schedule and adolescents’ obesity, overall and stratified by income level

	Crude model		Adjusted model 1^a^		Adjusted model 2^b^		Adjusted model 3^c^	
Mothers’ work schedule	OR	95% CI	OR	95% CI	OR	95% CI	OR	95% CI
Overall (*N* = 1743)								
Standard	1.00		1.00		1.00		1.00	
Nonstandard	**1.56**	**1.02–2.40**	1.47	0.94–2.28	1.49	0.95–2.34	1.44	0.92–2.27
Not employed	1.20	0.71–2.04	1.21	0.70–2.09	1.22	0.71–2.11	1.18	0.68–2.04
Stratified by income level								
Low–middle income (*N* = 621)								
Standard	1.00		1.00		1.00		1.00	
Nonstandard	0.93	0.49–1.74	0.97	0.50–1.86	0.97	0.50–1.90	0.88	0.40–1.74
Not employed	1.06	0.46–2.40	1.02	0.44–2.38	1.06	0.45–2.49	0.95	0.40–2.27
High income (*N* = 825)								
Standard	1.00		1.00		1.00		1.00	
Nonstandard	**2.80**	**1.43–5.50**	**2.83**	**1.41–5.69**	**2.93**	**1.45–5.92**	**2.90**	**1.43–5.86**
Not employed	1.59	0.69–3.66	1.90	0.81–4.50	1.89	0.80–4.48	1.83	0.77–4.37

^a^Adjusted for adolescent’s sex, adolescent’s type of high school, adolescent’s part-time job, mother’s age, mother’s psychological distress, household income, living with grandparents, and living with siblings

^b^Adjusted model 1 + father’s work schedule

^c^Adjusted model 2 + adolescent’s lifestyle habits

### Sensitivity analysis

Sensitivity analyses confirmed the robustness of the results. In the analyses presenting NSWS as five categories (Additional file 1), all work shifts were positively associated with adolescents’ obesity. Stratified by adolescents’ sex, similar associations were observed among both boys and girls, although the association with some work shifts did not reach a level of significance, probably due to the small sample size. In the analyses restricted to employed mothers (Additional file 2), after adjustment of mothers’ working hours, mothers’ NSWS was significantly associated with adolescents’ obesity only in high-income groups (OR: 2.80, 95% CI: 1.43–5.50), compared with mothers’ who had SWS.

## Discussion

This study demonstrated an adverse effect of maternal NSWS on obesity among Japanese adolescents aged 16–17 years in the Tokyo metropolitan area. This association was observed only in adolescents living in high-income families than in those living in low–middle income families. Adolescents from low–middle-income families had a higher prevalence of obesity, regardless of their mothers’ work schedules.

The association of mothers’ NSWS with adolescent obesity demonstrated in this study of Japanese participants is consistent with results from other developed countries [13, 15, 16]. We believe that our study has additional value as it expands on the generalizability of previous studies in children of later elementary [14–16] and junior high school ages [13] by demonstrating an effect in adolescents of high school age. Furthermore, this association was independent of mothers’ working hours. As already mentioned, many studies have suggested that long working hours of parents have adverse effects on childhood obesity [8, 9]. In our study, the NSWS of mothers were partially due to long working hours (above 40 h per week), but the significant association between mothers’ NSWS and adolescents’ obesity remained after adjustment of mothers’ working hours. This implies that maternal work schedules, as well as working hours per se, should be taken into account in the formulation of policy to prevent adolescent obesity.

We found an association between mothers’ NSWS and adolescents’ obesity only among adolescents living in higher-income households. This finding is inconsistent with the results of a previous study in 2008 [13], but supported by other studies that evaluated the association of mothers’ long working hours and childhood obesity [9, 26, 27]. These conflicting results may be explained by differences in the regional characteristics of study populations, such as urban or rural residence and area-level socioeconomic status [28]. Of note, our study was conducted only in the metropolitan area.

We propose the following explanations for the differential effect of maternal NSWS on adolescent obesity according to income status. In our sample, the prevalence of obesity among adolescents in low–middle income groups was high and did not differ with mothers’ work schedules. This may be because socioeconomic disadvantages per se, including low household income, is a risk factor for childhood obesity [29, 30]. Adolescents in low–middle income households may have poorer quality food intake than those in high-income households, due to less healthy food availability in the home and inadequate knowledge regarding healthy lifestyles [31]. In addition, although most Japanese adolescents rarely prepare their own meals [32], mothers in low–middle income households (irrespective of work schedules) may be less able to address their children’s diet quality and physical activity due to time pressure and inadequate access to healthy food, especially compared with mothers in high-income households [33]. Mothers with SWS in high-income households can spend more time preparing healthy meals than their counterparts with NSWS, which may contribute to a difference in the prevalence of obesity among adolescents in high-income groups associated with their mothers’ work schedules. In addition, compared to mothers with SWS in high-income households, the employment of mothers with NSWS is of lower status, which may lead to stress and, in turn, affect their adolescent children. Since gender segregation in the labor market is quite pronounced, and employment opportunities for women are limited in Japan [17], mothers have no choice but to work in poor quality employment at night or early in the morning when wages are higher to acquire additional household income [34].

However, the mechanisms behind the association between mothers’ NSWS and adolescents’ obesity remain uncertain. No clear patterns were evident between mothers’ work schedules and poor lifestyle habits when stratified by household income, and taking into account poor lifestyle habits in the logistic regression models did not explain the association between mothers’ NSWS and adolescents’ obesity. Unmeasured factors including nutritional balance, the frequency of eating snacks, and parents’ obesity may explain the association.

To our knowledge, this is the first study conducted using a relatively large population-based data set to demonstrate an association between mothers’ NSWS and prevalence of obesity among adolescents of high school age. However, our study had some limitations. First, the cross-sectional study design precluded us from establishing a causal relationship between maternal NSWS and adolescent obesity. However, the possibility of reverse causality – that is, adolescents’ obesity leads to mothers’ NSWS – was very unlikely. Second, response rates to our questionnaires were somewhat low (around 40%), and therefore our sample may not have been truly representative of the population as a whole, despite our efforts to conduct a broad-ranging survey. Third, we could not consider the duration of mothers’ NSWS work history because it was not measured in Survey of Child Living Conditions. Longer durations of mothers’ NSWS may have greater effects on adolescents’ obesity than shorter ones. Fourth, we could not assess the effect of unmeasured confounders, such as maternal occupation. Finally, the assessment of height and weight was based on adolescent self-reporting. Thus, we cannot eliminate the possibility of misclassification, although previous studies in Japan have shown a high degree of agreement between self-reported height and weight and their measured values [35].

Policy makers should consider mothers’ work schedules as well as mothers’ working hours in strategies for preventing child or adolescent obesity. It is important for mothers to be available during key times in children’s and adolescents’ days when they are not in school, including dinnertime, the post-dinner hours, waking time in general, and weekends. However, mothers in specific occupations, such as doctors and nurses, cannot avoid NSWS. To address this situation, some studies suggest that fathers’ involvement in unpaid household work may modify the impact of mothers’ NSWS on adolescent obesity [36]. Although this study did not focus on fathers, fathers need to be present during key times in their children’s and adolescents’ lives. However, there are still strong social perceptions of gender roles in Japan (i.e., the man is the breadwinner, while the woman is the caregiver) [17]. Approximately 70% of fathers do not commit to child care and unpaid household work on weekdays in Japan, and they rarely are involved in preparing healthy meals [37]. Therefore, fathers’ work schedules hardly contributed to adolescents’ obesity, as shown in Table 2. Therefore, policy makers should also consider the facilitation of fathers’ involvement in family life. Furthermore, it would be useful to enhance education for cooking skills and meal preparation habits for adolescents.

## Conclusions

Mothers’ NSWS was associated with obesity in adolescents of high school age in high-income families. Research on the effect of NSWS on child or adolescent obesity is still limited and further research is needed to replicate these findings prospectively in different ages and cultural contexts, and to clarify the mechanisms by which parental NSWS affects children’s or adolescents’ obesity. Given the importance of early prevention, as healthy habits are formed in early childhood, the impact of mothers’ continuous NSWS from early childhood on adolescent obesity should also be considered.

## Supplementary Information

**Additional file 1.** Association between mothers’ several types of nonstandard work schedule and adolescents’ obesity among a high-income group, overall and by sex.

**Additional file 2.** Association between mothers’ work schedule and adolescents’ obesity for employed mothers, overall and stratified by income level.

## Data Availability

The data that support the findings of this study are available from Tokyo Metropolitan Government Bureau of Social Welfare and Public Health but restrictions apply to the availability of these data, which were used under license for the current study, and so are not publicly available.

## Abbreviations

BMI: Body mass index
95% CI: 95% confidence intervals
IOTF: International Obesity Task Force
OR: Odds ratios
NSWS: Nonstandard work schedules
