# Supplementary material for: Mothers’ nonstandard work schedules and adolescent obesity: a population-based cross-sectional study in the Tokyo metropolitan area
Source: BMC Public Health. 2021 Jan 28;21:237. doi: 10.1186/s12889-021-10279-w (PMC7845102; doi:10.1186/s12889-021-10279-w)
Supplement: Supplementary file 1 — Additional file 1. Association between mothers’ several types of nonstandard work schedule and adolescents’ obesity among a high-income group, overall and by sex. [file 12889_2021_10279_MOESM1_ESM.docx]

| **Supplemental Table 1. Association between mothers’ several types of nonstandard work schedule and adolescents’ obesity** | | | | | | | | | | | |
| --- | --- | --- | --- | --- | --- | --- | --- | --- | --- | --- | --- |
| **among a high-income group, overall and by sex^a^** | | | |  |  |  |  |  |  |  |  |
|  |  | Overall (N = 825) | | | |  | Boy (N = 389) | |  | Girl (N = 436) | |
| Mothers’ NSWS | | N | (%) | OR | 95% CI |  | OR | 95% CI |  | OR | 95% CI |
| (1) Morning (5–8 am) |  |  |  |  |  |  |  |  |  |  |  |
|  | No | 617 | (74.8) | 1.00 |  |  | 1.00 |  |  | 1.00 |  |
|  | Yes | 31 | (3.8) | **9.20** | **3.65–23.19** |  | **7.87** | **2.00–30.82** |  | **23.13** | **5.03–106.38** |
|  | Not employed | 31 | (3.8) | 1.37 | 0.63–2.97 |  | 1.31 | 0.48–3.63 |  | 1.92 | 0.52–7.05 |
| (2) Night (8–10 pm) |  |  |  |  |  |  |  |  |  |  |  |
|  | No | 604 | (73.2) | 1.00 |  |  | 1.00 |  |  | 1.00 |  |
|  | Yes | 44 | (5.3) | **4.35** | **1.71–11.04** |  | **4.00** | **1.19–13.48** |  | **7.97** | **1.49–42.74** |
|  | Not employed | 177 | (21.5) | 1.24 | 0.58–2.69 |  | 1.28 | 0.47–3.54 |  | 1.56 | 0.45–5.47 |
| (3) Overnight (10 pm–5 am) |  |  |  |  |  |  |  |  |  |  |  |
|  | No | 628 | (76.1) | 1.00 |  |  | 1.00 |  |  | 1.00 |  |
|  | Yes | 20 | (2.4) | **5.26** | **1.63–17.01** |  | **10.13** | **1.80–57.17** |  | 4.59 | 0.71–29.54 |
|  | Not employed | 177 | (21.5) | 1.18 | 0.55– 2.53 |  | 1.21 | 0.44–3.35 |  | 1.42 | 0.42–4.87 |
| (4) Saturday |  |  |  |  |  |  |  |  |  |  |  |
|  | No | 415 | (50.3) | 1.00 |  |  | 1.00 |  |  | 1.00 |  |
|  | Yes | 233 | (28.2) | **2.54** | **1.28–5.03** |  | 1.84 | 0.76–4.48 |  | **4.81** | **1.40–16.55** |
|  | Not employed | 177 | (21.5) | 1.61 | 0.70–3.72 |  | 1.40 | 0.48–4.06 |  | 2.83 | 0.66–12.13 |
| (5) Sunday or holiday |  |  |  |  |  |  |  |  |  |  |  |
|  | No | 533 | (64.6) | 1.00 |  |  | 1.00 |  |  | 1.00 |  |
|  | Yes | 115 | (13.9) | **2.69** | **1.31–5.51** |  | 2.33 | 0.89–6.13 |  | **4.84** | **1.45–16.2** |
|  | Not employed | 177 | (21.5) | 1.37 | 0.62–3.02 |  | 1.35 | 0.48–3.78 |  | 2.00 | 0.54–7.42 |
| ^a^Adjusted for adolescent's sex, adolescent's type of high school, adolescent's part-time job, mother's age, mother's psychological distress, | | | | | | | | | | | |
| household income, living with grandparents, living with siblings, father’s work schedule, and lifestyle variables. | | | | | | | | | | |  |
