# Supplementary material for: Mothers’ nonstandard work schedules and adolescent obesity: a population-based cross-sectional study in the Tokyo metropolitan area
Source: BMC Public Health. 2021 Jan 28;21:237. doi: 10.1186/s12889-021-10279-w (PMC7845102; doi:10.1186/s12889-021-10279-w)
Supplement: Supplementary file 2 — Additional file 2. Association between mothers’ work schedule and adolescents’ obesity for employed mothers, overall and stratified by income level. [file 12889_2021_10279_MOESM2_ESM.docx]

| **Supplemental Table 2. Association between mothers’ work schedule and** | | | |
| --- | --- | --- | --- |
| **adolescents’ obesity for employed mothers, overall and stratified by income level^a^** | | | |
| Mothers’ work schedule | | OR | 95% CI |
| *Overall* (N = 1376) |  |  |  |
|  | Standard | 1.00 |  |
|  | Nonstandard | 1.41 | 0.88–2.26 |
| *Stratified by income level* |  |  |  |
| Low–middle income (N = 519) |  |  |  |
|  | Standard | 1.00 |  |
|  | Nonstandard | 1.11 | 0.54–2.28 |
| High income (N = 648) |  |  |  |
|  | Standard | 1.00 |  |
|  | Nonstandard | **2.56** | **1.23–5.32** |
| ^a^Adjusted for adolescent's sex, adolescent's type of high school, adolescent's employment status, | | | |
| mother’s age, mother’s psychological distress, household income, living with grandparents, | | | |
| living with siblings, father's work schedule, life style variables, and mother’s work hours. | | | |
